# Challenging T > MIC Using Meropenem vs. *Escherichia coli* and *Pseudomonas aeruginosa*

**DOI:** 10.3389/fphar.2022.840692

**Published:** 2022-04-01

**Authors:** A. Nussbaumer-Pröll, S. Eberl, E. Kurdina, L. Schmidt, M. Zeitlinger

**Affiliations:** Department of Clinical Pharmacology, Medical University of Vienna, Vienna, Austria

**Keywords:** %T>MIC, meropenem, *P. aeruginosa*, *E. coli*, resistance, TKC

## Abstract

**Objective:** For meropenem 40%T > MIC is associated with optimal killing of *P. aeruginosa* and *E. coli*. However, it is unknown how the distribution of %T > MIC through a treatment day impacts the antimicrobial effect *in vitro*. Therefore, we investigated the *in vitro* antibiotic activity of meropenem, precisely if 40%T > MIC is achieved in one single long period (single dose), 2 × 20% periods (dosing-bid), or 3 × 13.3% (dosing t.i.d.) thereby keeping the overall period of T > MIC constant.

**Material/Methods:** Time kill curves (TKC) with *P. aeruginosa*-ATCC-27853 and *E. coli*-ATCC-25922 and five clinical isolates each were implemented over 24 h in CAMHB with concentrations from 0.25×MIC-32×MIC. Periods over and under MIC were simulated by centrifugation steps (discarding supernatant and refilling with fresh CAMHB). Double and triple dosing involved further addition and removal of antibiotic. Complementary growth controls (GC) with and without centrifugation steps were done and the emergence of phenotypical resistance was evaluated (repeated MIC-testing after antibiotic administration).

**Results:** No impact of centrifugation on bacterial growth was seen. TKC with *P. aeruginosa* showed the best killing in the triple dosage, followed by the double and single dose. In multiple regimens at least a concentration of 4×MIC was needed to achieve a recommended 2-3 log10 killing. Likewise, a reduction of *E. coli* was best within the three short periods. Contrary to the TKCs with *P. aeruginosa* we could observe that after the inoculum reached a certain CFU/mL (≥10^8), no further addition of antibiotic could achieve bacterial killing (identified as the inoculum effect). For *P. aeruginosa* isolates resistance appeared within all regimens, the most pronounced was found in the 40%T > MIC experiments indicating that a single long period might accelerate the emergence of resistance. Contrary, for *E. coli* no emergence of resistance was found.

**Conclusion/Outlook:** We could show that not solely the %T > MIC is decisive for an efficient bacterial eradication *in vitro*, but also the distribution of the selected %T > MIC. Thus, dividing the 40%T > MIC in three short periods requested lowers antibiotic concentrations to achieve efficient bacterial killing and reduces the emergence of resistance in *P. aeruginosa* isolates. The distribution of the %T > MIC did impact the bacterial eradication of susceptible pathogens *in vitro* and might play an even bigger role in infections with intermediate or resistant pathogens.

## Introduction

In antimicrobial therapy the efficacy of antibiotics is not only dependent on the drug itself, but also on the patient’s physiology including disease state, comorbidities or age, and the variety of the bacterial species (Maguigan et al., 2021).

Antimicrobial resistance is only one aspect which impacts treatment success. Especially during the COVID-19 pandemic many people had to be treated in intensive care units and were vulnerable to secondary infections, e.g., with multi-drug resistant microbes (Pelfrene et al., 2021). Thus, last line antibiotics such as meropenem, are administered. Resistance data of meropenem for Germany, which is also representative for Austria, is listed in the Paul-Ehrlich-Gesellschaft (PEG)-S2k guideline “Calculated parenteral initial treatment of bacterial infections: Microbiology.” The current resistance prevalence for meropenem against clinical isolates is given in percentage: *Enterobacteriaceae*, especially *Klebsiella pneumoniae* (∼1%); *Pseudomonas aeruginosa* (15–17%); *Acinetobacter baumannii* (∼29.5%), and methicillin resistant *Staphylococcus aureus* (MRSA) (11.8–13.5%) (Kresken et al., 2020).

Two important aspects for a successful antibiotic treatment are the unbound antibiotic concentration at the target site and the effect the drug has on the bacterial pathogen, which is best explained by pharmacokinetic (PK) and pharmacodynamic (PD) indices. The three most important PK-PD indices are the time over the MIC (T > MIC), the peak drug concentration over MIC (Cmax/MIC), and the 24 h area under the concentration curve over MIC (AUC/MIC) ratios (Nicolau, 2001; Frimodt-Moller, 2002; Toutain et al., 2002; Rodríguez-Gascón et al., 2021). Different PK-PD indices are set as targets for achieving antimicrobial efficacy of different antibiotic classes, e.g., for time dependent beta-lactam antibiotics it is the %T > MIC (Rodríguez-Gascón et al., 2021).

For intensive care unit (ICU) patients it might be beneficial to target a free drug concentration of a beta-lactam of 100%T > MIC or even 100%T > 4×MIC, whereas in patients with normal renal and hepatic function the serum drug concentrations should exceed the MIC of the causative pathogen for at least 40–70% of the dosing interval (Tam et al., 2002; McKinnon et al., 2008; Abdul-Aziz et al., 2020; Ďuricová et al., 2020; Tan et al., 2021).

However, four main concerns arise when using threshold values for PK/PD parameters.

First, the PK/PD threshold target seems to vary between different beta-lactam antibiotics as described by Ambrose. et al. and Craig, which has been summarized in Table 1 (Craig, 1997; Craig, 1998; Ambrose et al., 2007; Masich et al., 2018).

**TABLE 1:** A summary of the range of the percentage time above the minimal inhibitory concentration (%T > MIC) for carbapenems, penicillins, and cephalosporins for Gram-positive and Gram negative pathogens is depicted (Craig, 1997; Craig, 1998; Ambrose et al., 2007).

Pathogen	Carbapenems (%T > MIC)	Penicillins (%T > MIC)	Cephalosporins (%T > MIC)
Gram positive	20–30	30–40	40–50
Gram negative	40–50	50–60	60–70

Second, the PK/PD threshold might vary depending on the magnitude of effect that is targeted, e.g., for more serious infections a 2-3 log reduction in CFU/mL might be targeted (e.g., hospital acquired pneumonia treated with quinolones), while for others bacteriostasis might be sufficient (e.g., complicated skin and skin structure infections treated with tigecycline) (Ambrose et al., 2007). Third, the MIC as the PD part of the threshold equally suffers from several limitations. The MIC provides only limited information on the kinetics of the drug action (e.g., the rate of bactericidal activity and whether increasing antimicrobial concentrations can enhance this rate) and it depends on the number of bacteria on a single time point (Mueller et al., 2004; Friberg, 2021).

Fourth, the PK/PD indices only insufficiently consider the shape of the concentration time curve of the antibiotic. Thereby, a high Cmax/MIC ratio might or might not be accompanied by a high AUC/MIC value. Likewise for T > MIC all concentrations below the MIC are equally ineffective and similar to all concentrations above the MIC are treated as equally effective. However, concentrations that are just below the MIC might show some anti-infective activity compared to others close to zero and concentrations slightly above the MIC might not display the maximum effect that is only achieved with higher concentrations (Mueller et al., 2004; Friberg, 2021).

Studies such as from Dandekar et al. have already proven that treatment success with meropenem might be optimized by prolonged infusion and 3 times daily doses (0.5 and 2 g meropenem every 8 h, 3 h infusion) (Dandekar et al., 2003). Nevertheless, it is unknown whether the distribution of the percentage T > MIC through a treatment day might impact *in vitro* antimicrobial activity over 24 h.

**FIGURE 1:**
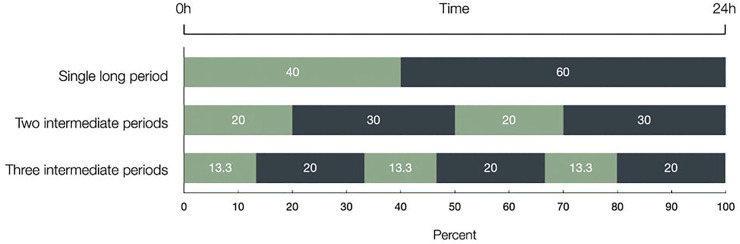
The three different distributions of the 40%T > MIC within 24 h are depicted and given in percentage. Light bars represent the time meropenem was present and dark bars represent antibiotic free time. The 40%T > MIC has been achieved in one single long period, in two intermediate periods (2 × 20%), and in three intermediate periods (3 × 13.3%), thereby keeping the overall period of T > MIC constant. Time over Minimal Inhibitory Concentration (T > MIC).

For meropenem 40%T > MIC is usually associated with optimal killing of *P. aeruginosa* and *E. coli* (Nicolau, 2008). Thus, to better understand the mechanism behind the well-established %T > MIC parameter, we set out to investigate the antibiotic activity of meropenem, precisely if the 40%T > MIC is achieved in one single long period (single dose), two 20% periods (dosing bid), or 3 × 13.3% (dosing t.i.d.) thereby keeping the overall period of T > MIC constant. Figure 1 explains this experimental setting in detail.

## Materials and Methods

### Bacterial Strains

Reference strains were obtained from the American Type Culture Collection (ATCC); *P. aeruginosa* ATCC-27853 and *E. coli* ATCC-25922. Five bacterial isolates of *P. aeruginosa* and five bacterial isolates of *E. coli* collected from blood cultures were provided by the Department of Microbiology of the General Hospital in Vienna.

### Antibiotic

For susceptibility testing and pharmacodynamic experiments meropenem (trihydrate powder, Sigma-Aldrich, Germany), dissolved in dimethyl sulfoxide (DMSO), was used.

### Liquid Growth Media

Cation adjusted Mueller Hinton Broth (CAMHB) (Sigma-Aldrich, Germany) was used as liquid growth media containing 17.5 g/L casein acid hydrolysate, 3 g/L beef extract, 1.5 g/L starch, 20–25 mg/L calcium, and 10–12.5 mg/L of magnesium with a pH of 7.3 ± 0.2.

### Solid Growth Media

Columbia agar plates (bioMérieux, Marcy-I’Etoile, France) containing 5% sheep blood were used as solid growth media for *P. aeruginosa* and *E. coli* strains.

### Broth Microdilution

Broth microdilution for evaluating the minimal inhibitory concentration (MIC) of the test strains was done according to the performance standards for antimicrobial susceptibility testing of the Clinical and Laboratory Standards Institute (CLSI) (National Committee for Clinical Laboratory Standards). The approximate concentration range was set based on EUCAST ranges for *P. aeruginosa* 0.008–2 mg/L and *E. coli* 0.008–0.06 mg/L.

### Time Kill Curves

All TKC analyses and growth controls (GC) were performed in triplicate over 24 h in a shaking water bath (amplitude 22 mm, 150 amplitudes/min) at 37°C under aerobic conditions. The bacterial suspension was adjusted to 1.5 × 10^8 CFU/ml in NaCl, corresponding to a McFarland standard of 0.5, 100 µL was added to the test tubes filled with CAMHB to a final volume of 10 ml. After 1 h of pre-incubation, 100 µl aliquots were taken of each falcon tube to determine the CFU/mL at time point 0 h before the addition of the antibiotic. The samples were pipetted in the first row of a 96-well microtiter plate. Subsequently, seven serial dilution steps with a volume of 20 µL were carried out in the 96-well microtiter plates filled with 180 µL of 0.9% NaCl in rows two to seven. Aliquots of 20 µL of each concentration were dropped onto Columbia blood agar plates and incubated at 37°C under aerobic conditions for 24 h. This procedure was also done for subsequent time points.

From the meropenem stock dilutions were made (freshly prepared before every dosing) to achieve final concentrations several fold above and below the respective MIC of the pathogens (0.25×MIC to 32×MIC) by always adding 100 µL of the stock to the 14-ml tubes. To simulate 40%T > MIC, 2 × 20%, and 3 × 13.3%T > MIC centrifugation steps at 37°C for 5 min at 1300 g were done at certain time points to create antibiotic free time. The supernatant was discarded, falcon tubes were refilled with fresh CAMHB and vortexed to resuspend the bacterial pellet. Double and triple dosing involved further addition of antibiotic. Complementary GCs with and without centrifugation steps were done.

In the 40%T > MIC setting after 9.6 h the samples were centrifuged to create a meropenem free time. The CFU/mL was determined at 0, 2, 9.6, and 24 h. Underlined time points indicate meropenem administration and bold time points represent the centrifugation time points.

To simulate 2 × 20%T > MIC centrifugation of the tubes was done after 4.8 h after each antibiotic administration. Samples were taken after 0, 2, **4.8**, 12, 14, **16.8,** and 24 h.

Finally, to achieve 3 × 13.3%T > MIC the removal of meropenem was done after 3.2 h after the antibiotic administration. Aliquots were drawn after 0, 2, **3.2**, 8, 10, **11.2**, 16, 18, **19.2,** and 24 h.

For penems, a bactericidal concentration is commonly defined as a 3 log10 reduction in cell counts within 12–24 h. Thus, we set a threshold of 2-3 log10 reduction of CFU/mL in our experiments to better compare the impact of distributing the 40%T > MIC on bacterial killing through a treatment day.

### Emergence of Phenotypical Resistance

The emergence of phenotypical resistance was evaluated by repeated MIC testing after antibiotic administration. Thus, up to 3 CFU from the Columbia agar plates of 0 and 24 h of each tested concentration and of all three dosing regimens were collected with a sterile Q-tip and broth microdilution was performed as mentioned above. Ratios of the evaluated MIC of 24 and 0 h were calculated to evaluate a potential emergence of resistance.

## Results

### Growth and TKC

No impact of centrifugation on bacterial growth of all bacterial strains was seen.

**FIGURE 2:**
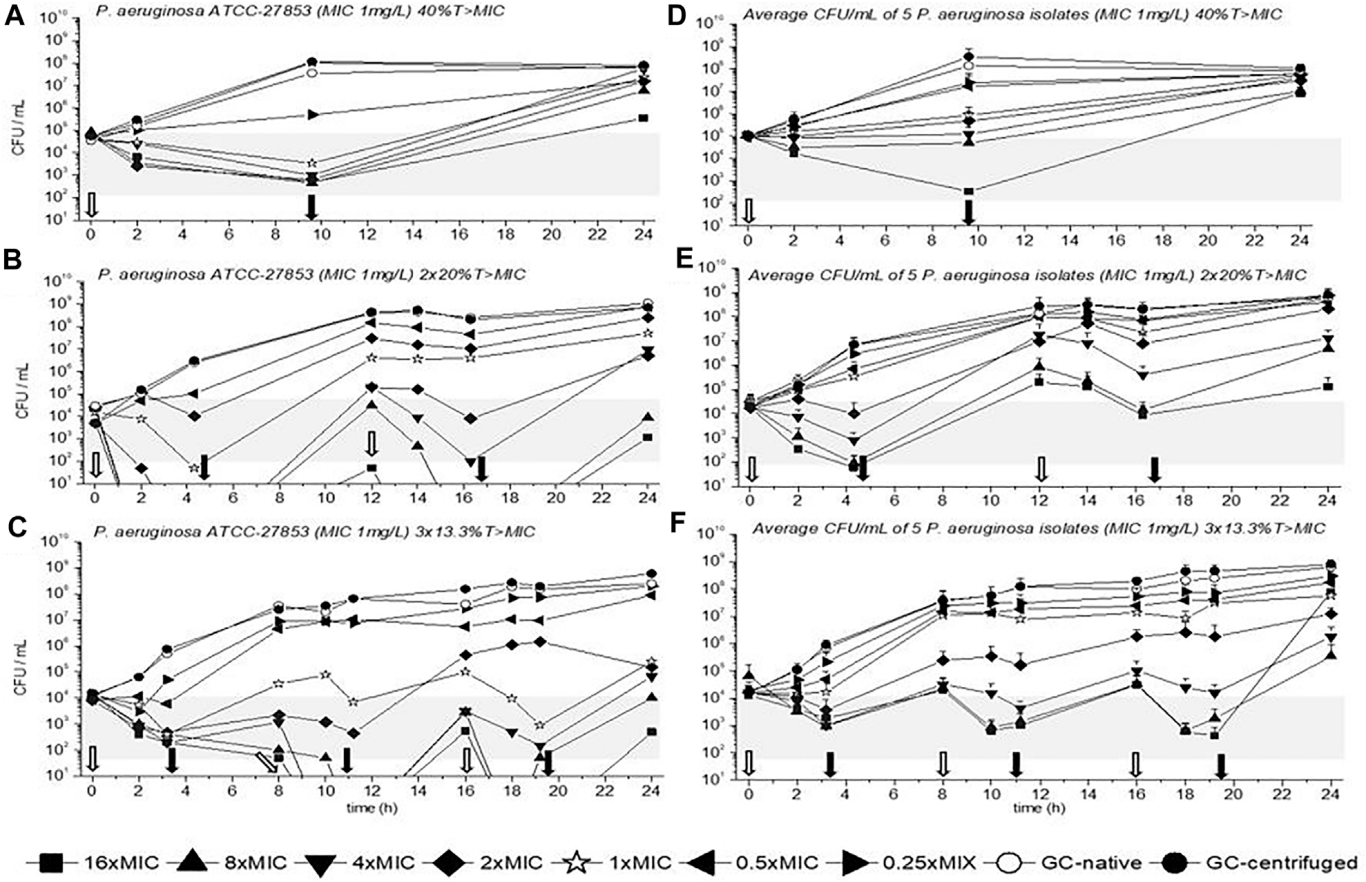
Time kill curves (TKC) with meropenem of *P. aeruginosa* ATCC-27853 **(A)**, **(B)**, **(C)** and of the average CFU/mL of five *P. aeruginosa* isolates **(D)**, **(E)**, **(F)** are shown over 24 h with standard deviation. In **(A)**, **(B),** and **(C)** the standard deviations are very small and overlaid by symbols. The circular symbols represent the growth controls; filled show the centrifuged and empty depict the native growth control (GC). The concentration of the minimal inhibitory concentration (MIC) of the tested strains is given as a star symbol. Empty arrows indicate antibiotic addition and filled arrows indicate antibiotic removal.

TKC with *P. aeruginosa* ATCC-27853 are depicted in Figures 2A,B,C for 40%, 2 × 20%, and 3 × 13.3%T > MIC. The average CFU/mL of all five *P. aeruginosa* isolates is also shown in Figures 2D,E,F for all three dosing regimens. Furthermore, empty arrows indicate antibiotic addition and filled arrows indicate antibiotic removal.

In all settings the concentration of 1×MIC could slow down bacterial growth or even reduce the initial bacterial count until the first antibiotic removal. Overall, best killing over 24 h was achieved in the three short periods with 3 × 13.3%, followed by the 2 × 20%T > MIC and the 40%T > MIC for ATCC-27853 and the clinical isolates. In multiple regimens at least a concentration of 4×MIC was needed to achieve a 2-3 log10 killing which was defined as the threshold according to the prescribing information (gray bars in Figure 2). Regrowth was present in all dosing regimens, especially in the 40%T > MIC setting. The most pronounced regrowth was seen in Figure 2F in the triple dosage with 16×MIC (filled square) between the last antibiotic removal and the 24-h time point, probably driven by selected mutants.

**FIGURE 3:**
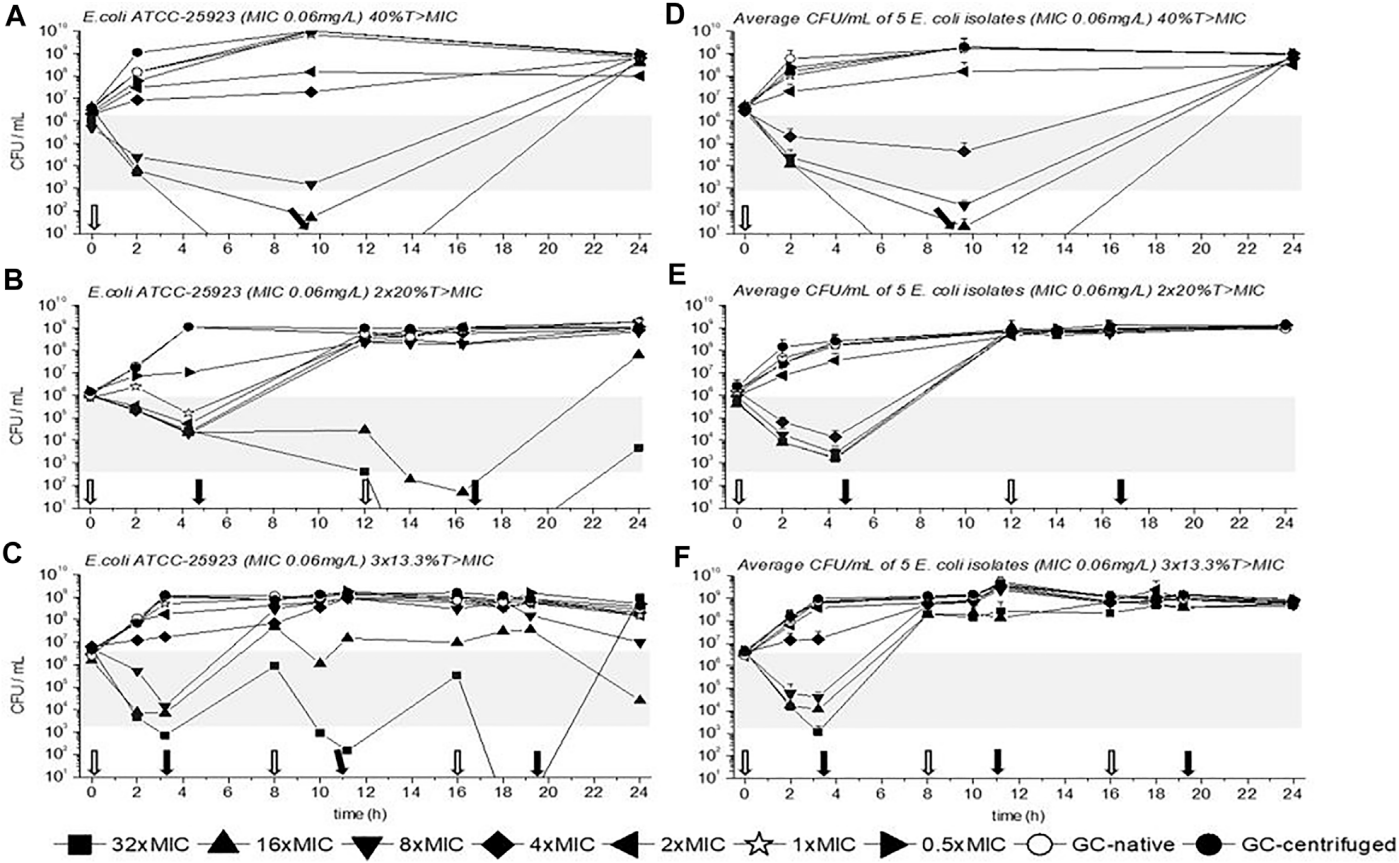
Time kill curves (TKC) with meropenem of *E. coli* ATCC-25923 **(A)**, **(B)**, **(C)** and of the average CFU/mL of five *E. coli* isolates **(D)**, **(E)**, **(F)** are shown over 24 h with standard deviation. In **(A)**, **(B),** and **(C)** the standard deviations are very small and overlaid by symbols. The circular symbols represent the growth controls, filled show the centrifuged, and empty depict the native GC. The concentration of the MIC of the tested strains is given as a star symbol. Empty arrows indicate antibiotic addition and filled arrows indicate antibiotic removal.

TKC with *E. coli* ATCC-25923 is depicted in Figures 3A,B,C for 40%, 2 × 20%, and 3 × 13.3%T > MIC. The average CFU/mL of all five *E. coli* isolates are also shown in Figures 3D,E,F for all three dosing regimens. Moreover, as in Figure 2 empty arrows indicate antibiotic addition and filled arrows indicate antibiotic removal.

Contrary to *P. aeruginosa* experiments a concentration of 1×MIC did not reduce bacterial growth within the first dosing interval (until the first removal of the antibiotic), at least a concentration of 4×MIC was needed. Nevertheless, similar to the previous data with *P. aeruginosa* strains a reduction of *E. coli* ATCC-25923 within the first interval of dosing was best with the triple dosage compared to the other dosing regimens. Thus, as mentioned before a concentration of 4×MIC or higher achieved a 2-3 log10 killing (gray bars in Figure 2). Regrowth of *E. coli* ATCC-25923 and of all *E. coli* isolates was observed after antibiotic removal, depending on the time the antibiotic was present. In detail, the longer the antibiotic was present the later antibiotic free time was generated, and the later regrowth occurred. Furthermore, contrary to the TKCs with *P. aeruginosa* isolates we could observe that after the inoculum reached a certain CFU/mL (10^8 or higher), no exposure to antibiotic could achieve bacterial killing. This was identified as the inoculum effect, which was confirmed in additional experiments (data not shown).

Another possibility to evaluate the bacterial killing of the three different experimental settings is the comparison of CFU/mL at the start and at the end of the experiment. Applying a log10 on the ratio of CFU/mL of 24 h and CFU/mL of 0 h for the tested concentrations results in either positive or negative values in the different settings. Positive values indicate bacterial growth and negative bacterial killing. For *P. aeruginosa* ATCC-27853 in the 40%T > MIC experiment a value of 3.2, in the 2 × 20%T > MIC experiment a value of 2.7, and in the 3 × 13.3%T > MIC experiment a value of 0.7 was calculated, confirming the best growth inhibition in the three short periods with a concentration of 4×MIC. Moreover, the highest reduction of CFU/mL was achieved with a concentration of 16×MIC with values of 0.8 in the 40%T > MIC, -0.6 in the 2 × 20%T > MIC, and -1.5 in the 13.3%T > MIC experiments, confirming again best killing in the three short periods. Contrary to *E. coli* ATCC-25923, the concentration of 4×MIC could not reduce the CFU/mL in any setting, as values of 2.2, 2.9, and 2.5 were obtained for 40%, 2 × 20%, and 3 × 13.3%, respectively.

### Resistance

The median ratios of the MIC values (24/0 h) are depicted for *P. aeruginosa* and *E. coli* isolates in Tables 2 and 3, respectively. A ratio at 1 or below indicates no change of the MIC. Ratios above 1 indicate an elevation of the MIC. Within MIC evaluation deviations of 1-2-fold dilutions are common, thus only ratios above 2 were rated as emergence of phenotypical resistance. For *P. aeruginosa* isolates the emergence of phenotypical resistance was present within all regimens. Concentrations at 1×MIC or higher in the 40%T > MIC setting showed up to 18-fold higher MIC values indicating that single administration favors emergence of resistance compared to the other dosing regimens for *P. aeruginosa* isolates. Contrary, for *E. coli* no emergence of resistance was found.

**TABLE 2:** The median ratios of the minimal inhibitory concentration (MIC) from 24 to 0 h are depicted for *P. aeruginosa* isolates for all three regimens and all tested concentrations. A ratio at 1 or below indicates no change of the MIC. Ratios above 2 indicate an emergence of resistance.

*P. aeruginosa* isolates							
Median ratio 24h/0 h	16×MIC	8×MIC	4×MIC	2×MIC	1×MIC	0.5×MIC	0.25×MIC
40%	2	3	18	5	6	8	1
2 × 20%	2	1.5	8	2	1	1	1
3 × 13.3%	1	2	2.25	2.5	6	8	1

**TABLE 3:** The median ratios of the minimal inhibitory concentration (MIC) from 24 to 0 h are depicted for *E. coli* isolates for all three regimens and all tested concentrations. A ratio at 1 or below indicates no change of the MIC. Ratios above 2 indicate an emergence of resistance.

*E. coli* isolates							
Median ratio 24h/0 h	32×MIC	16×MIC	8×MIC	4×MIC	2×MIC	1×MIC	0.5×MIC
40%	1	1	1	1	1	1	1
2 × 20%	1	1	1	1	1	1	1
3 × 13.3%	0.5	1	0.75	0.75	0.5	0.5	0.5

## Discussion

According to the current paradigm, the most important PK-PD parameter for beta-lactam antibiotics, such as meropenem, is the percentage of time it exceeds the MIC (Steffens et al., 2021). Our TKC results could confirm that not solely the %T > MIC is decisive for an efficient bacterial eradication *in vitro*, but also the distribution of the selected %T > MIC. Our data indicate that three short periods of 13.3%T > MIC, which equals 3 × 3.2 h, above a concentration of at least 4×MIC of meropenem achieved best bacterial killing within 24 h compared to the continuous 40%T > MIC, which equals a period of 1 × 9.6 h.

Previous studies have already shown that, for example, in critically ill patients the %T > MIC for beta-lactams might need to be adjusted from 40–70%T > MIC to 100%T > MIC to meet the target attainment (Maguigan et al., 2021). Moreover, Nielsen et al. outlined in a predictive semi-mechanistic PK/PD model how changes in MIC of the target pathogen or alternating renal clearance rates of the patient could shift PK/PD indices of benzylpenicillin, cefuroxime, erythromycin, and other antibiotics (Nielsen et al., 2011). For cefuroxime they state bacteriostatic activity and bactericidal activity is best achieved with 30%T > MIC and 41%T > MIC, respectively. Yet, they could show in their simulation that in treatment of patients with reduced renal clearance or displaying a pathogen with a 2× higher MIC, the T > MIC is no longer the best option, within these cases the AUC/MIC seems to be best for target attainment (Nielsen et al., 2011; Friberg, 2021).

Despite these relevant considerations we raised another question; what happens if the recommended 40%T > MIC of meropenem against *P. aeruginosa* and *E. coli* isolates is not present in one single long period but distributed over the treatment day, thereby keeping the overall period of T > MIC constant?

This was clearly shown for *P. aeruginosa* ATCC-27853, the five clinical isolates of *P. aeruginosa,* and for *E. coli* ATCC-25922. In detail, within the 3 × 13.3% experiments the bacterial count could be kept low (2-3 log10 reduction of the initial inoculum) for a longer time (up to 20 h) compared to the continuous 40%T > MIC (up to 9.6 h). One explanation could be that the bacteria are more often and more evenly exposed to meropenem during the 24 h. Namely every 8 h for 3.2 h in the 3 × 13.3% setting compared to once for 9.6 h in the continuous 40%T > MIC. Therefore, the bacteria might not be able to adapt that fast on the presence of meropenem compared to the continuous setting. In the single long period 14.4 h of antibiotic free time is generated between antibiotic removal and the end of the experiment. Contrary, in the three short periods there are only 4.8 h of antibiotic free time between antibiotic removal and the next antibiotic administration. Thus, recurrent short antibiotic exposure of 3 × 13.3% seems to eradicate the bacteria over the 24 h better than the continuous 40%T > MIC as less selection pressure on the bacteria might be present and adapted resistances of the pathogens to survive meropenem exposure might not evolve that rapidly. This has been discussed as well by Baker at al., as they state that the presence of antibiotics creates a selection pressure for antibiotic resistant microbes, and large populations of bacteria are more likely to harbor drug resistance than small populations (Baker et al., 2018).

In TKCs with the *E. coli* isolates strong regrowth was found after antibiotic removal in every setting achieving CFU/mL of 10^8 or higher and no further antibiotic exposure could induce bacterial killing, which we could identify as the inoculum effect in separate experiments. One explanation why regrowth was so strong in *E. coli* experiments might be the higher initial inoculum of ∼10^6 CFU/ml compared to *P. aeruginosa* experiments which was between 10^4 and 10^5 CFU/ml. Nevertheless, regrowth was present in all experimental settings after antibiotic removal.

Dividing the 40%T > MIC in three dosing intervals did not only achieve efficient bacterial killing but it reduced as well the emergence of resistance in *P. aeruginosa* isolates.

In the continuous 40%T > MIC experiments *P. aeruginosa* isolates displayed a median MIC of 18 mg/L at a concentration of 8×MIC compared to 2.25 mg/L in the 3 × 13.3%T > MIC setting. Again, this might be due to the longer time *P. aeruginosa* isolates were exposed to meropenem in the continuous 40%T > MIC experiments, introducing a higher selective pressure and therefore bacterial strains might evolve resistant mechanisms.

This is highlighted by the fact that the MIC values obtained directly after the dosing interval of 9.6 h of concentrations 4×MIC already showed at least threefold higher MIC values (data not shown).

Nevertheless, the study has several limitations. First, we did not test how 100%T > MIC, sometimes recommended for critical settings, would have impacted killing and the emergence of resistances.

Moreover, additional sampling time points within all experiments would have been an advantage to further compare killing and growth between the different settings. However, since the experiments had to run day and night for 24 h it has been hampered by feasibility.

Another critical point to mention is the washing procedure as it has been performed only by one centrifugation step, discarding old CAMHB and resuspending the bacterial pellet with new CAMHB. Thus, additional washing with CAMHB, PBS, or NaCl would have been assured to decrease the risk of residual antibiotic in the samples.

Furthermore, we only performed phenotypical evaluation of resistance and did not investigate potential genotypical mechanisms.

Last, we wanted to test our hypothesis of distributing %T > MIC in an extreme setting, by adding and removing antibiotic through centrifugation steps creating an all or nothing situation instead of a simulation dedicated to clinically used dosing regimens.

## Conclusion and Outlook

In conclusion we could show that not solely the %T > MIC is decisive for an efficient bacterial eradication *in vitro*, but also the distribution of the selected %T > MIC. Thus, dividing the 40%T > MIC in three dosing intervals required lower antibiotic concentrations to achieve efficient bacterial killing and reduced the emergence of resistance in *P. aeruginosa* isolates. The distribution of the %T > MIC did impact the bacterial eradication of susceptible pathogens and might play an even bigger role in infections with intermediate or resistant pathogens.

Hence, this study shows that defined PK-PD targets such as the T > MIC should be examined more closely and the shape of the concentration vs. time curve deserves more attention in future investigations. Subsequent studies should be performed to challenge Cmax/MIC and AUC/MIC values in comparable settings.

## Data Availability

The original contributions presented in the study are included in the article/Supplementary Material, further inquiries can be directed to the corresponding author.
